# Systemic treatment for brain metastasis in HER2- positive advanced breast cancer: what have we learned so far?

**DOI:** 10.18632/oncotarget.28435

**Published:** 2023-07-07

**Authors:** Marta Vaz Batista, José Perez-Gracia, Inês Eiriz, Maria Gion, Antonio Llombart, Sofia Braga, Javier Cortés

**Keywords:** brain metastasis, HER2+ breast cancer

The better survival of Human Epidermal growth factor receptor-type 2 positive (HER2+) breast cancer (BC) patients unmasked the biological predilection of this BC subtype for development of brain metastasis (BM). Indeed, central nervous system (CNS) is a frequent metastatic site for HER2+ advanced BC patients. Over the last years, new therapeutic strategies targeting the HER2 protein have been introduced for systemic treatment of HER2+ BC - either tyrosine kinase inhibitors, monoclonal antibodies, or antibody-drug conjugates. Patients with BMs have a poorer outcome, compared with patients without BMs, but their prognosis is also improving with the introduction of new anti HER2+ - targeted therapies.

The majority of drugs targeting HER2+ were not specifically evaluated for patients with BM. In fact, historically patients with baseline BMs were excluded from clinical trials. In the CLEOPATRA trial, in testing the role of double blockade with trastuzumab and pertuzumab in combination with docetaxel as first line therapy for metastatic HER2+ BC, baseline BMs were not allowed [1], but more recent trials permitted inclusion of patients with treated and asymptomatic BMs. In these, brain activity can be inferred by subgroup analysis [2–5]. The efficacy is mainly reported as median overall survival, median progression free survival, overall response rate, CNS progression, time to CNS intervention or clinical benefit rate in the included patients with BM, evaluated by Response Evaluation Criteria in Solid Tumours (RECIST) criteria. However, this approach might lack sensibility to predict CNS efficacy of these drugs. Lapatinib was one of the first drugs to specifically be investigated in patients with untreated BMs from BC or even in patients with progressive BMs after local therapy. More recently, a phase III trial with tucatinib also included patients with active BMs [6]. Besides this broader inclusion criteria, another important shift is the use of Response Assessment in Neuro-Oncology (RANO) criteria for intracranial activity assessment. RANO also takes into account the use of corticosteroids and clinical status, which might be more sensitive than RECIST for CNS evaluation.

Our group has been working in the DEBBRAH trial, using trastuzumab deruxtecan for different settings of CNS involvement: stable or progressing BM and/or leptomeningeal carcinomatosis. We included patients with HER2+ and HER2-low BC. The final results are yet to be reported, but so far, we observed intracranial responses in HER2+ BC patients [7, 8]. Activity of trastuzumab-deruxtecan in patients with HER2+ BC and untreated or progressing after local therapy BMs also has been shown in another phase II trial [9].

With the accumulating evidence on the activity of HER2-targeted therapy in BM from HER2+ BC, the next question to be raised is whether these drugs can be used for BMs delay or prevention. Supporting this possibility is the impressive results of HER2CLIMB, with an estimated 1-year CNS-progression free survival of 53.3% in the tucatinib arm and 0% in the control arm for the subgroup of patients with stable BMs [6].

With these new drugs now available in clinical practice, we expect HER2+/HER2-low BC patients with BMs to live longer and to live better.
